# 320-row CT transcatheter aortic valve replacement planning with a single reduced contrast media bolus injection

**DOI:** 10.1371/journal.pone.0204145

**Published:** 2018-09-13

**Authors:** Daddy Mata-Mbemba, Aissam Labani, Soraya El Ghannudi, Mi-Young Jeung, Patrick Ohlmann, Catherine Roy, Mickaël Ohana

**Affiliations:** 1 Department of Diagnostic Imaging, Hospital for Sick Children, Toronto, Ontario, Canada; 2 Department of Diagnostic Imaging, Nouvel Hôpital Civil–Strasbourg University Hospital, Strasbourg, France; 3 Department of Cardiology, Nouvel Hôpital Civil–Strasbourg University Hospital, Strasbourg, France; 4 iCube Laboratory, University of Strasbourg, Illkirch, France; Universita degli Studi di Roma La Sapienza, ITALY

## Abstract

**Objective:**

To reduce the iodine load required for CT Transcatheter Aortic Valve Replacement (TAVR) planning on a 320-row scanner by acquiring the two CT TAVR steps (ECG-gated aortic root CTA and non-gated aorto-ilio-femoral CTA) within a single contrast media bolus injection.

**Methods:**

50 consecutive patients (82.6±6.9 years; 56% female) were prospectively enrolled and underwent a TAVR planning using a 320-row CT, with ECG-gated aortic root CTA immediately followed by a non-gated aorto-iliac acquisition, all within a single bolus of 40-70mL of Iohexol 350mgI/mL. The Iodine load, image quality, SNR, CNR and radiation dose were compared using a Mann-Whitney test to that of 24 consecutive patients (84.3±4.8 years, 58% female) previously imaged on a 64-row scanner with a conventional two-step protocol.

**Results:**

Iodine load was reduced by 44%. All examinations were of diagnostic quality, with improvement of the aortic root CTA image quality (4.9±0.3 versus 4.6±0.5, *p*<0.01) and a non-significant decrease of the aorto-iliac CTA image quality (4.7±0.6 versus 4.9±0.3, *p* = 0.07). SNR and CNR were significantly improved in the aortic root CTA (14.0±5.3 and 10.4±4.5 versus 10.3±4.2 and 6.8±3.3, p<0.01 for both) and non-significantly higher in the aorto-iliac CTA (16.5±8.0 and 14.1±7.9 versus 14.7±5.5 and 12.5±5.0, *p* = 0.42 and *p* = 0.66). Total radiation dose was reduced by 32%.

**Conclusion:**

320-row CT scanner enables a 44% reduction of iodine load in TAVR planning, while maintaining excellent aorto-ilio-femoral arterial enhancement and lowering radiation dose.

## Introduction

Aortic stenosis is the third most prevalent cardiovascular disease worldwide [1], with a poor natural prognosis since the survival rate of untreated patients with symptomatic severe stenosis is 60% at 1 year and 32% at 5 years [2]. Transcatheter Aortic Valve Replacement (TAVR) is now established as a valid alternative therapeutic procedure [3, 4] for patients who either have contraindications to open surgery or decline it [5]. Computed Tomography Angiography (CTA) plays an essential role in the selection of patients suitable for this technique, being the reference method for the aortic annulus and aortic root sizing, for the assessment of the coronary ostia position and for the prediction of the appropriate projection angles in the angiography suite [6–8]. CTA is also essential in defining iliac vessels tortuosity, stenosis, and severity of calcified atherosclerosis, to plan the safest access route and minimize vascular complications [9]. Using a 64 or a 128-row single source scanner, a comprehensive TAVR planning protocol commonly requires two consecutive steps: a retrospectively ECG-gated aortic root CTA first, followed by an ungated aorto-ilio-femoral CTA [10, 11]. For each of both steps, a bolus injection of 30 to 90mL of iodine contrast media (CM) is required, bringing the total amount of CM delivered up to 180mL. This represents a major limitation in an elderly population with frequently impaired renal function [12]. Therefore, various strategies have been described through the literature to reduce to the strict minimum the quantity of iodine CM required for CT TAVR planning.

Wide area-detector CT have the ability to acquire ECG-gated examinations in a single volume acquisition, and this–combined with the fast gantry rotation of the second-generation 320-detector row CT scanners (275msec)–enables the acquisition of a cardiac CT in less than a second, compared to more than 10 seconds on 64-row scanners [13]. We hypothesize that we can take advantage of this technology to reduce the iodine load required for CT TAVR planning on a 320-row scanner by acquiring the two CT TAVR steps (ECG-gated aortic root CTA and non-gated aorto-ilio-femoral CTA) within a single contrast media bolus injection.

## Materials and methods

This study was performed at a single university hospital, after Institutional Review Board approval (ethics board of the "*Hôpitaux Universitaires de Strasbourg*"). Written informed consent was obtained from all participants in the study group. For the retrospective analysis of the examinations of the control group, the need of an informed consent was waived by our Institutional Review Board.

### Patients

From May 2013 to April 2014, all patients referred to our radiology department for a TAVR planning were prospectively and consecutively included to constitute our study group. An indication for TAVR, *i*.*e*. a severe aortic valve stenosis with contra-indication to or refusal of open surgery, was therefore the only inclusion criterion. The exclusion criteria were a severe renal impairment with an estimate glomerular filtration rate (eGFR) < 20 mL/min and a proven allergy to iodine contrast media.

A control group was constituted by the retrospective inclusion of all patients who underwent a TAVR planning on a 64-row scanner from March 2012 to March 2013. These consecutive patients were acquired using a conventional two-step approach with reinjection.

### CT imaging

All patients in the study group were examined using a second-generation 320-row CT scanner (Aquilion ONE Vision Edition, Toshiba Medical Systems, Otawara, Japan).

First, the aortic root CTA was acquired in volume mode using a retrospective ECG-gated acquisition and the following CT parameters: 16cm width, 100kV, gantry rotation time of 0.275s, auto-mA maxed at 300, acquisition over 1 heartbeat.

Immediately afterwards (*i*.*e*. with a delay ranging from 3 to 6 seconds due to table motion), a non-gated aorto-ilio-femoral CTA was acquired in helical mode from the mandibular angle down to the mid-thigh using the following parameters: 100kV, gantry rotation time of 0.275s, pitch of 0.813, auto-mA maxed at 400.

Both (aortic root and aorto-ilio-femoral CTA) acquisitions were obtained within a single breath hold and after a single bolus injection of Iohexol 350mg/mL (Omnipaque, General Electric Healthcare, Milwaukee, Wisconsin), using an automatic power injector at a rate of 3.5mL/sec, followed by 30mL of saline chaser at a rate of 3mL/sec. Patients with a Body Mass Index (BMI) lower than 23 kg/m^2^ had a 40-50mL bolus, patients with a BMI between 23 and 30 had a 60-70mL bolus and patients with a BMI over 30 had a 70-80mL bolus. The acquisition was triggered using a bolus-tracking technique with a Region of Interest (ROI) positioned in the descending thoracic aorta and a 180 Hounsfield Units (HU) threshold.

All patients from the control group were examined with a 64-row scanner (CT750HD, General Electric Healthcare, Milwaukee, Wisconsin).

The aortic root CTA was performed first, using a retrospective ECG-gating (100kV, 0.35sec rotation time, auto-mA maxed at 700) and a bolus of 60-70mL of Iomeprol 350 to 400mg/mL (Iomeron, Bracco, Milan, Italy) injected at a rate of 4mL/sec. A bolus-tracking technique with a ROI in the descending thoracic aorta and a 200 Hounsfield Units (HU) threshold was used.

Afterwards, the aorto-ilio-femoral CTA was acquired from the mandibular angle down to the mid-thigh (non-gated helical acquisition, 100kV, gantry rotation time of 0.35sec, pitch of 0.984, auto-mA maxed at 450), using an additional bolus of 30 to 90mL (30-50mL for patients with BMI lower than 25kg/m^2^, 60–90 for BMI equal or higher than 25) of Iomeprol 350 to 400mg/mL injected at a rate of 3.5mL/sec. The ROI for the bolus tracking technique was placed within the descending thoracic aorta and a 160HU threshold was used. For both injections, 30mL of saline chaser at 3mL/sec were added after the iodine CM bolus.

The CT acquisitions in both groups of patients were reconstructed with a soft kernel and a third-generation iterative reconstruction algorithm: Adaptive Iterative Dose Reduction using 3 Dimensional (AIDR-3D) in the “standard” setting for the study group [13], and Adaptive Statistical Iterative Reconstruction set at 50% for the control group.

In both group, the aortic root volume was reconstructed every 10% from 0 to 90%.

No β-blockers or other heart-rate lowering agents were used for both groups.

### Qualitative CT evaluation

All acquisitions from both the study and the control groups were anonymized and randomized. Aortic root and aorto-iliac CTA were independently reviewed by two radiologists (MO and AL, with 7 and 5 years of experience in cardiovascular CT, respectively) using a dedicated workstation (Vitrea version 6.4, Vital Images, USA). All the examinations were analyzed once in multiplanar reconstructions. Images were displayed in a vascular window setting (level 100HU and width 900HU); the radiologist could adjust the window display settings.

For the aortic root CTA, the readers examined all reconstructions from 0 to 90%, with a special focus on the systolic 20–40% volumes that are used for the annulus sizing [14]. The quality of the ECG gating, the sharpness of the aortic wall, the clear depiction of the calcifications and the valve leaflets and the identification of the annulus plane were the criterion that had to be assessed by the readers.

For the aorto-iliac CTA, the level of luminal arterial enhancement, the sharpness of the arterial wall, the amount of image noise and the clear depictions of the supra-aortic trunks and the iliac arteries calcifications were the points evaluated by the readers. Significant (*i*.*e*. greater than 50%) iliac stenoses were documented.

Based on these specific points, the two radiologists independently evaluate both acquisitions with a five-point Likert scale (Table 1).

**Table 1:** Description of the five-point likert scale used in qualitative analysis of CTA.

Score	Diagnostic value	Description	Clinical value
1	Uninterpretable examination	Absent opacification, extremely severe artifacts	Non-diagnostic
2	Poor image quality	Insufficient opacification, major artifacts	
3	Acceptable image quality	Sufficient opacification, minor artifacts	Diagnostic
4	Good image quality	Excellent arterial opacification, negligible artifacts	
5	Excellent image quality	Excellent arterial opacification, no artifacts	

### Quantitative CT evaluation

For each patient, the total dose (in gram) of iodine CM injected and the Dose Length Product (DLP) expressed in mGy.cm for each acquisition were recorded.

The effective dose of radiation in mSv was obtained by multiplying the DLP by the specific conversion coefficient of 0.0144 mSv/mGy.cm for the aortic root CTA and 0.0141 mSv/mGy.cm for the aorto-iliac CTA [13, 15].

Another radiologist (DMM, with 10 years of experience in cardiovascular CT) measured the vascular attenuations in HU and their standard deviations at different levels on the aortic root and aorto-iliac CTA using circular regions of interest (ROI) averaging at least 5 mm^2^. For the aortic root CTA, these ROI were placed in the aortic sinus, the sino-tubular junction and the ascending portion of the aorta. For the aorto-iliac CTA, they were placed in the tubular portion of the ascending aorta, in the abdominal aorta at the level of the renal arteries, in the right or left common iliac artery and in an erector spinae muscle avoiding fatty tissue infiltration. For each location, the measurements were repeated three times. Mean vascular attenuation and mean noise, corresponding to the mean standard deviation, were averaged for the arterial measurements and used to compute the signal-to-noise ratio (SNR), which was obtained by dividing the mean vascular attenuation by the mean noise, and the contrast-to-noise ratio (CNR), which was calculated using the following formula as described by Yuan et al [16]:

$$CNR=\frac{mean\;vascular\;attenuation–muscle\;attenuation}{mean\;noise}$$

### Statistics

All statistical analyses were performed by using software JMP Pro version 10 (SAS Institute Inc., Cary, North Carolina, USA).

Data are presented as means ± standard deviations unless specified otherwise.

A Student *t* test was used to compare the demographic data of the two groups of patients.

The interobserver agreement between the two readers regarding subjective image quality assessment was evaluated with the Cohen κ test.

A Mann-Whitney U test was used to compare the score in image quality, the dose of iodine CM, the dose of radiation exposure, the SNR and the CNR between both groups.

A *p* value below 0.05 was considered statistically significant.

## Results

### Patients

Patients’ demographics and main clinical characteristics are shown in Table 2.

**Table 2:** General characteristics of patients.

Parameters	Study Group (n = 50)	Control Group (n = 24)	*p* value
**Age**	82.6 (±6.9)	84.3 (±4.8)	0.21
**Sex**			0.85
Male	22 (44%)	10 (42%)	
Female	28 (56%)	14 (58%)	
**BMI**			0.89
Mean	26.2 (±5.5)	26.1 (±5.2)	
Range	17.6–40.8	16.7–34.8	
**Diabetes** *(type I and II)*	11 (22%)	6 (25%)	0.78
**Hypertension**	32 (64%)	17 (71%)	0.56
**Extracardiac arteriopathy**	14 (28%)	8 (33%)	0.65
**Severely impaired renal function** *(eGFR<50ml/min)*	28 (54%)	11 (45%)	0.42
**Heartrate during CT acquisition**			0.63
Mean	81.2 (±18.4)	79.3 (±21.1)	
Range	51–130	55–121	

59 patients were approached, and 56 accepted to be included in the study. 5 were excluded due to severe renal impairment (eGFR < 20mL/min) and 1 patient was excluded because of history of severe allergy to CM, leaving 50 patients (mean age: 82.6±6.9 years; range:61–96, 56% female) who constituted our study group.

Twenty four consecutive patients were retrospectively included in the control group.

There were no statistically significant differences in age (*p* = 0.21), sex ratio (*p* = 0.85) nor BMI (*p* = 0.89) between both groups.

### Qualitative CT evaluation

The image quality scores of the aortic root CTA was significantly better in the study group (mean: 4.9±0.3, range: 3.5–5, κ = 0.39) than in the control group (4.6±0.5; range: 3.5–5; κ = 0.32) (*p* = 0.0004).

No statistically significant difference was found between image quality scores of the aorto-iliac CTA between the study (4.7±0.6, range: 3–5, κ = 0.61) and the control (4.9±0.3, range: 3.5–5; κ = 0.30) groups (*p* = 0.07). In addition, the amount of calcified plaque in the iliac arteries (90% in the study group and 92% in the control group) and the frequency of greater than 50% stenosis in the iliac arteries (26% in the study group and 25% in the control group) were comparable between both groups (*p* = 0.82 and *p* = 0.93, respectively).

**Fig 1:**
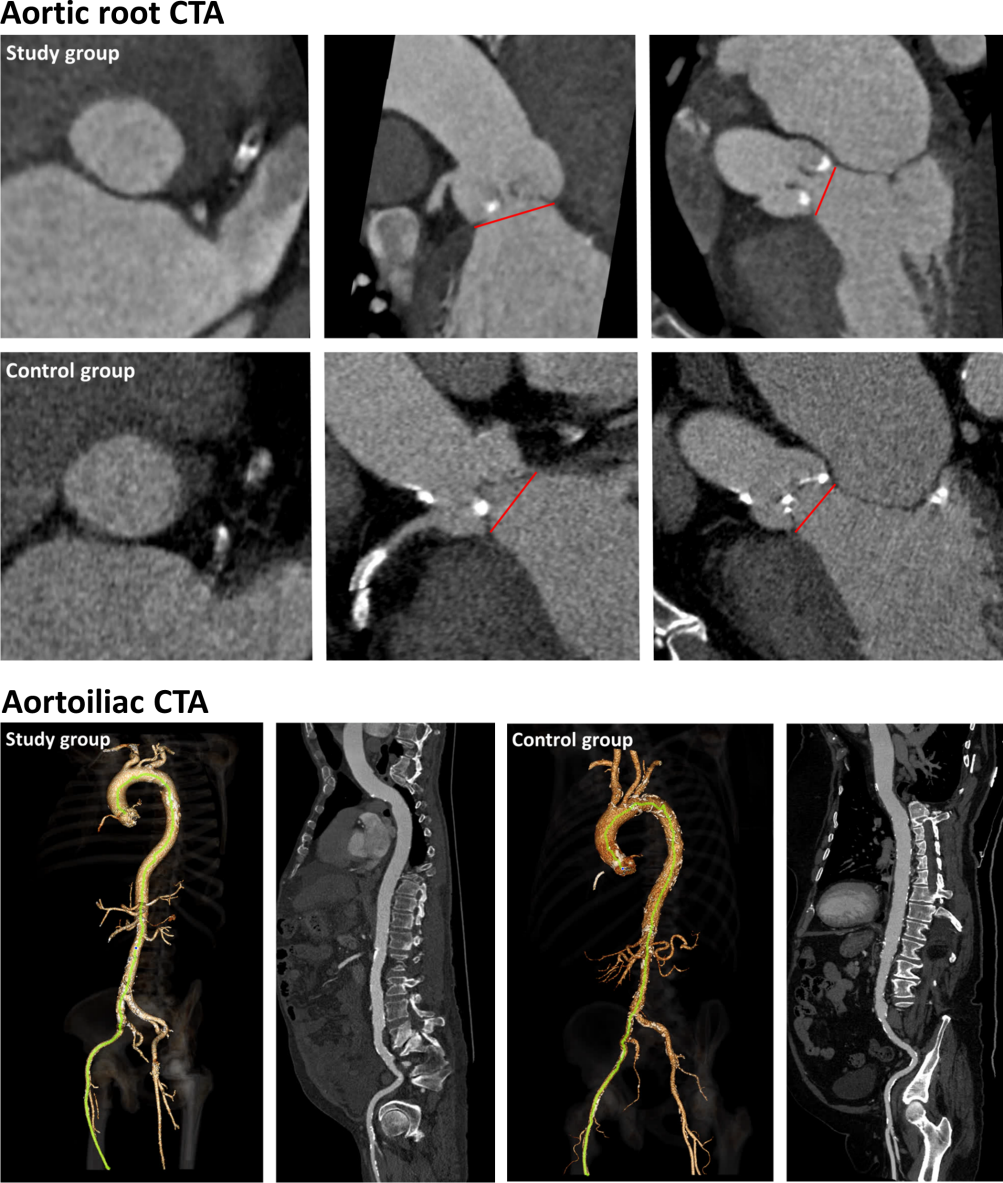
Comparative example of image quality. Up is the aortic root CTA, depicting the aortic annulus in axial and frontal planes. Down is the aortoiliac CTA in 3D and curved mutliplanar reconstructions. Study group images are from a 76 years-old man from the study group, whose BMI was 32.5, and were obtained after a single bolus injection of 70mL of Iohexol 350mgI/ml. Total radiation dose was 935mGy.cm. Control group images are from a 86 years-old man from the control group, with a BMI of 30.1, and were obtained after a 2 steps acquisition protocol using a total amount of 120mL of Iohexol 350mgI/ml. Total radiation dose was 1653 mGy.cm.

Demonstrative examples are shown in Fig 1.

### Quantitative CT evaluation

#### Dose of iodine CM

The total iodine load was significantly lower in the study group (23.6±3.4 g, range: 14–31.5, corresponding to a median volume of 67mL) than in control group (43.3±8 g, range: 28–63, corresponding to a median volume of 124mL), which represented a reduction of 44% (*p*<0.0001).

#### Radiation dose

The radiation dose was significantly lower in the study group for the ECG-gated aortic root CTA (565±432 mGy.cm versus 800±249 mGy.cm in the control group; *p* = 0.0007) as well as for the aorto-ilio-femoral CTA (518±253 mGy.cm versus 785±184 mGy.cm in the control group; *p*<0.0001). Consequently, the total dose received by the patient during the whole examination was lowered in the study group by around 32% (1083±615 mGy.cm versus 1585±350 mGy.cm in the control group; *p* = 0.0002). When translated in effective dose, it represents a mean radiation dose of 15.4±8.7 mSv in the study group versus 22.6±4.9 mSv in the control group.

#### SNR and CNR

For the ECG-gated aortic root, the SNR in the study group (14.0±5.3) was significantly higher than in the control group (10.3±4.2; *p* = 0.0013). The CNR was also significantly higher in the study group (10.4±4.5) than in the control group (6.8±3.3; *p* = 0.0004). In details, the mean arterial enhancement was higher than 300HU in 90% of the study group versus 75% of the control group, and between 150 and 300HU in 10% and 25%, respectively.

For the aorto-iliac CTA, no significant difference in SNR (study group: 16.5±8.0 versus control group: 14.7±5.5; *p* = 0.42) nor in CNR (study group: 14.1±7.9 versus control group: 12.5±5.0; *p* = 0.66) were noted in both groups. In details, the mean arterial enhancement was higher than 300HU in 60% of the study group versus 79% of the control group, between 150 and 300HU in 34% and 21%, respectively; and lower than 150HU in 6% and 0%, respectively.

Details about arterial attenuation are given in Table 3.

**Table 3:** Detailed HU attenuation within the aortic root and the aortoiliac CTA.

Measures	Study Group (n = 50)	Control Group (n = 24)	*p* value
**Aortic root**			
HU attenuation	480 ±139 (169–925)	372 ±114 (183–629)	<0.01
SD	36 ±6 (24–49)	38 ±8 (24–54)	0.21
SNR	14 ±5.3 (4.2–31.3)	10.3 ±4.2 (4.4–20.6)	<0.01
CNR	10.4 ±4.5 (2.7–25.8)	6.8 ±3.3 (2.2–14.2)	<0.01
**Aortoiliac CTA**			
HU attenuation	364 ±170 (117–828)	400 ±95 (221–595)	0.16
SD	23 ±10 (14–55)	28 ±4 (20–37)	<0.01
SNR	16.5 ±8 (5.1–36.9)	14.7 ±5.5 (7.3–25.1)	0.42
CNR	14.1 ±7.9 (3.3–34.3)	12.5 ±5 (2.2–14.2)	0.66

Numbers are given in a *mean ±SD (minimum–maximum)* fashion.

## Discussion

We have demonstrated in this study that the second generation 320-row CT could achieve a complete TAVR CT workup with acquisition of the ECG-gated aortic root followed by the aorto-ilio-femoral CTA within one single iodine CM bolus injection, resulting in a significant (44%) reduction of the total iodine load, with a mean iodine dose of 23.6g.

This significant reduction of the total iodine load is beneficial in the clinical setting, since one to two thirds of patients screened for TAVR have chronic kidney disease, and therefore a theoretically increased risk of contrast induced nephropathy, associated with an increased morbidity and mortality [17, 18]. One of the most effective precaution to prevent this complication is to decrease the total dose of iodine CM to the lowest possible [19]. As described below, a number of authors [20–25] have studied different acquisition protocols to achieve this goal.

One of these techniques is to use the ultra-high pitch mode (pitch higher than 3) available on dual-source scanners. They allow acquisition of the whole aorta with freezing of the aortic root within one single reduced CM bolus injection [20], which results in significant reduction of iodine dose (to as low as 10,5 g) and radiation dose (down to 250 mGy.cm) [20][21]. However, an accurate assessment of the aortic root annulus maximum size that is critical for TAVR planning is not possible by lack of retrospective ECG gating associated of these techniques.

Others used the wide volume acquisitions available on wide-area detector CT. This technique acquires the whole aorta with sequential ECG-gated volume acquisition covering up to 16cm at once [22], and consequently requires only one step and one bolus injection of CM. Although the image quality is excellent, the acquisition time remains lengthy (up to 25 seconds) and therefore the iodine load cannot be substantially reduced.

Dual energy CT provides Iodine density images and low keV reconstructions which maximize iodine contrast, therefore allowing a significant dose reduction to achieve a similar contrast uptake [23]. This technique has been used in the setting of TAVR planning almost to halve the iodine load required for the aorto-ilio-femoral CTA (30mL only) in case of a standard dual-step protocol [24]. However, the overall reduction in iodine load is not that significant since up to 70mL of CM are still required for the aortic root ECG-gated CTA.

As compared to aforementioned reports, we succeeded to significantly reduce the total dose of iodine CM. This successful experience have prompted us to implement the use of this single contrast media bolus injection TAVR protocol whenever a complete aorta CTA with excellent depiction of the ascending aorta is needed, including in patients with aortic aneurysm or dissection, or in the postoperative follow-up of ascending aorta surgery.

In this study, we also confirmed that the image quality of the ECG-gated aortic root CTA is significantly increased when using a second generation 320-row CT in comparison to 64-row CT. This appears consistent as the second generation 320-row CT prevents stair-step or misalignment artifacts since it uses a one-beat volume acquisition [26–28]. More interestingly, as high quality images are mandatory for better planning of vascular access routes, one of the concern was whether lowering the dose of CM would affect the quality of the aorto-ilio-femoral CTA. Our results show that when using a single injection and reduced dose of iodine CM, we do not significantly lose in image quality, SNR or CNR during this part of the examination. In addition, we have confirmed that the radiation dose in each of the two steps are significantly reduced with 320-row CT. If the decrease in not surprising for the aortic root CTA, due to the use of a volume acquisition [29], the significant decrease in the aorto-ilio-femoral helical acquisition is probably due to the use of more advanced acquisition optimizations and new generation detectors.

We should acknowledge some limitations to our study.

First, the concordance analysis of qualitative data showed only fair to moderate agreement between the two readers. This result might be explained by: (i) the high number of categories (five) as more categories lead to lower concordance, and (ii) the fact that the disagreement was in more than 90% of cases between class 4 and 5 (*i*.*e*. good versus excellent image quality), which does not affect the diagnosis of these patients.

Second, there are relatively many variables considered in this study (e.g. CT scanner platform, number of rows, iterative reconstruction methods, contrast volume, contrast bolus protocol) which could affect some of our results. So to alleviate effects of some cofounders, dedicated studies comparing 320-detector row imaging with two different contrast administration protocols prospectively, one dual-phase and the other single-phase, with corresponding differences in contrast volume administration, or two different scanner platforms with the same contrast protocol is recommended.

Third, given that there are relatively fewer 320-detector row CT scanners in routine practice in the world so far, this may affect the generalizability of our results.

Fourth, we were not able to evaluate the cardiac output (*i*.*e*. the left ventricular ejection fraction) for each patient, since the whole LV wasn’t systematically included in the retrospective ECG-gated acquisition. Consequently, one could speculate that patients with low cardiac output could experience a reduced image quality for the aorto-iliac acquisition when using a single injection protocol.

Fifth, despite the absence of a statistically significant difference, there were still 3 patients in our study group were the iliac attenuation was weak (i.e. below 150HU), rendering the evaluation of the arterial lumen challenging. The reason for this is probably multifactorial, and could be related to an intrinsic limitation of the single bolus protocol (insufficient amount of contrast media, late timing) or to the patient itself (low cardiac output, obesity). Future studies might be needed to better determine in which patients this single bolus protocol might not be appropriate, regarding the peripheral arterial evaluation.

Finally, the incidence of acute kidney injury could not be evaluated because most patients underwent a pre-operative coronarography 48 hours before or after the CT TAVR. Therefore, the follow-up creatinine dosage is not representative of the detrimental effect of the CT TAVR alone.

## Conclusion

The use of a 320-row CT scanner with single contrast media bolus injection acquisition protocol can halve the iodine load in TAVR planning, while maintaining excellent aorto-ilio-femoral arterial enhancement and significantly lowering the radiation dose.

This new approach could be useful whenever a complete aorta CTA with excellent depiction of the ascending aorta is needed.

## Supporting information

S1 Table Dataset for the 320-row group. (XLSX)

S2 Table Dataset for the 64-rows group. (XLSX)

## References

[1] Maganti K, Rigolin VH, Sarano ME, Bonow RO. Valvular heart disease: diagnosis and management. Mayo Clinic proceedings. 2010;85(5):483–500. Epub 2010/05/04. 10.4065/mcp.2009.0706 ; PubMed Central PMCID: PMC2861980. 20435842 PMC2861980

[2] Zajarias A, Cribier AG. Outcomes and safety of percutaneous aortic valve replacement. Journal of the American College of Cardiology. 2009;53(20):1829–36. Epub 2009/05/16. 10.1016/j.jacc.2008.11.059 . 19442881

[3] Smith CR, Leon MB, Mack MJ, Miller DC, Moses JW, Svensson LG, et al Transcatheter versus surgical aortic-valve replacement in high-risk patients. The New England journal of medicine. 2011;364(23):2187–98. Epub 2011/06/07. 10.1056/NEJMoa1103510 . 21639811

[4] Kodali SK, Williams MR, Smith CR, Svensson LG, Webb JG, Makkar RR, et al Two-year outcomes after transcatheter or surgical aortic-valve replacement. The New England journal of medicine. 2012;366(18):1686–95. Epub 2012/03/27. 10.1056/NEJMoa1200384 . 22443479

[5] Holmes DR Jr., Mack MJ, Writing C. Transcatheter valve therapy: a professional society overview from the American College of Cardiology Foundation and the Society of Thoracic Surgeons. Ann Thorac Surg. 2011;92(1):380–9. Epub 2011/07/02. 10.1016/j.athoracsur.2011.05.067 . 21718887

[6] Wuest W, Anders K, Schuhbaeck A, May MS, Gauss S, Marwan M, et al Dual source multidetector CT-angiography before Transcatheter Aortic Valve Implantation (TAVI) using a high-pitch spiral acquisition mode. European radiology. 2012;22(1):51–8. 10.1007/s00330-011-2233-0 . 21845463

[7] Leipsic J, Wood D, Manders D, Nietlispach F, Masson JB, Mayo J, et al The evolving role of MDCT in transcatheter aortic valve replacement: a radiologists' perspective. AJR American journal of roentgenology. 2009;193(3):W214–9. Epub 2009/08/22. 10.2214/AJR.08.2230 . 19696262

[8] Soon J, Pibarot P, Blanke P, Ohana M, Leipsic J. Multimodality Imaging for Planning and Follow-up of Transcatheter Aortic Valve Replacement. The Canadian journal of cardiology. 2017;33(9):1110–23. 10.1016/j.cjca.2017.03.024 . 28666614

[9] Lejay A, Caspar T, Ohana M, Delay C, Girsowicz E, Ohlmann P, et al Vascular access complications in endovascular procedures with large sheaths. The Journal of cardiovascular surgery. 2016;57(2):311–21. Epub 2015/11/26. . 26603161

[10] Holmes DR Jr., Mack MJ, Kaul S, Agnihotri A, Alexander KP, Bailey SR, et al 2012 ACCF/AATS/SCAI/STS expert consensus document on transcatheter aortic valve replacement. Journal of the American College of Cardiology. 2012;59(13):1200–54. Epub 2012/02/04. 10.1016/j.jacc.2012.01.001 . 22300974

[11] Achenbach S, Delgado V, Hausleiter J, Schoenhagen P, Min JK, Leipsic JA. SCCT expert consensus document on computed tomography imaging before transcatheter aortic valve implantation (TAVI)/transcatheter aortic valve replacement (TAVR). Journal of cardiovascular computed tomography. 2012;6(6):366–80. 10.1016/j.jcct.2012.11.002 . 23217460

[12] Thomsen HS, European Society of Urogenital R. European Society of Urogenital Radiology guidelines on contrast media application. Current opinion in urology. 2007;17(1):70–6. Epub 2006/12/05. 10.1097/MOU.0b013e328011c96f . 17143114

[13] Chen MY, Shanbhag SM, Arai AE. Submillisievert median radiation dose for coronary angiography with a second-generation 320-detector row CT scanner in 107 consecutive patients. Radiology. 2013;267(1):76–85. Epub 2013/01/24. 10.1148/radiol.13122621 ; PubMed Central PMCID: PMC3606544. 23340461 PMC3606544

[14] Jurencak T, Turek J, Kietselaer BL, Mihl C, Kok M, van Ommen VG, et al MDCT evaluation of aortic root and aortic valve prior to TAVI. What is the optimal imaging time point in the cardiac cycle? European radiology. 2015;25(7):1975–83. Epub 2015/02/25. 10.1007/s00330-015-3607-5 ; PubMed Central PMCID: PMC4457917. 25708961 PMC4457917

[15] Halliburton SS, Abbara S, Chen MY, Gentry R, Mahesh M, Raff GL, et al SCCT guidelines on radiation dose and dose-optimization strategies in cardiovascular CT. Journal of cardiovascular computed tomography. 2011;5(4):198–224. Epub 2011/07/05. 10.1016/j.jcct.2011.06.001 ; PubMed Central PMCID: PMC3391026. 21723512 PMC3391026

[16] Yuan R, Shuman WP, Earls JP, Hague CJ, Mumtaz HA, Scott-Moncrieff A, et al Reduced iodine load at CT pulmonary angiography with dual-energy monochromatic imaging: comparison with standard CT pulmonary angiography—a prospective randomized trial. Radiology. 2012;262(1):290–7. Epub 2011/11/16. 10.1148/radiol.11110648 . 22084206

[17] Bruce RJ, Djamali A, Shinki K, Michel SJ, Fine JP, Pozniak MA. Background fluctuation of kidney function versus contrast-induced nephrotoxicity. AJR American journal of roentgenology. 2009;192(3):711–8. Epub 2009/02/24. 10.2214/AJR.08.1413 . 19234268

[18] Mack MJ, Brennan JM, Brindis R, Carroll J, Edwards F, Grover F, et al Outcomes following transcatheter aortic valve replacement in the United States. Jama. 2013;310(19):2069–77. Epub 2013/11/19. 10.1001/jama.2013.282043 . 24240934

[19] Davenport MS, Cohan RH, Ellis JH. Contrast media controversies in 2015: imaging patients with renal impairment or risk of contrast reaction. AJR American journal of roentgenology. 2015;204(6):1174–81. Epub 2015/03/03. 10.2214/AJR.14.14259 . 25730301

[20] Bischoff B, Meinel FG, Reiser M, Becker HC. Novel single-source high-pitch protocol for CT angiography of the aorta: comparison to high-pitch dual-source protocol in the context of TAVI planning. The international journal of cardiovascular imaging. 2013;29(5):1159–65. Epub 2013/01/22. 10.1007/s10554-013-0182-1 . 23334190

[21] Marwan M, Achenbach S, Hell M, Schuhbaeck A, Gauss S, Bittner D, et al Third generation dual source Computed Tomography for evaluation of patients prior to transcatheter aortic valve replacement: comprehensive imaging with an ultra low-dose of contrast agent. Journal of the American College of Cardiology. 2015;65(10_S). 10.1016/S0735-1097(15)61263-3

[22] Li Y, Fan Z, Xu L, Yang L, Xin H, Zhang N, et al Prospective ECG-gated 320-row CT angiography of the whole aorta and coronary arteries. European radiology. 2012;22(11):2432–40. Epub 2012/06/05. 10.1007/s00330-012-2497-z . 22661055

[23] Ohana M, Jeung MY, Labani A, El Ghannudi S, Roy C. Thoracic dual energy CT: acquisition protocols, current applications and future developments. Diagnostic and interventional imaging. 2014;95(11):1017–26. Epub 2014/05/02. 10.1016/j.diii.2014.01.001 . 24780370

[24] Dubourg B, Caudron J, Lestrat JP, Bubenheim M, Lefebvre V, Godin M, et al Single-source dual-energy CT angiography with reduced iodine load in patients referred for aortoiliofemoral evaluation before transcatheter aortic valve implantation: impact on image quality and radiation dose. European radiology. 2014;24(11):2659–68. 10.1007/s00330-014-3263-1 . 24962826

[25] Pulerwitz TC, Khalique OK, Nazif TN, Rozenshtein A, Pearson GD, Hahn RT, et al Very low intravenous contrast volume protocol for computed tomography angiography providing comprehensive cardiac and vascular assessment prior to transcatheter aortic valve replacement in patients with chronic kidney disease. Journal of cardiovascular computed tomography. 2016;10(4):316–21. 10.1016/j.jcct.2016.03.005 ; PubMed Central PMCID: PMC4958553. 27061253 PMC4958553

[26] Dewey M, Zimmermann E, Deissenrieder F, Laule M, Dubel HP, Schlattmann P, et al Noninvasive coronary angiography by 320-row computed tomography with lower radiation exposure and maintained diagnostic accuracy: comparison of results with cardiac catheterization in a head-to-head pilot investigation. Circulation. 2009;120(10):867–75. Epub 2009/08/26. 10.1161/CIRCULATIONAHA.109.859280 . 19704093

[27] Rybicki FJ, Otero HJ, Steigner ML, Vorobiof G, Nallamshetty L, Mitsouras D, et al Initial evaluation of coronary images from 320-detector row computed tomography. The international journal of cardiovascular imaging. 2008;24(5):535–46. Epub 2008/03/28. 10.1007/s10554-008-9308-2 . 18368512

[28] Shuman WP, Green DE, Busey JM, Ramos MM, Branch KR, Koprowicz KM, et al Wide-detector axial CT versus 4 cm detector helical CT for transcatheter aortic valve replacement: iodine dose, radiation, and image quality. Clinical imaging. 2016;40(6):1213–8. 10.1016/j.clinimag.2016.08.002 . 27616154

[29] Hsiao EM, Rybicki FJ, Steigner M. CT coronary angiography: 256-slice and 320-detector row scanners. Current cardiology reports. 2010;12(1):68–75. Epub 2010/04/29. 10.1007/s11886-009-0075-z ; PubMed Central PMCID: PMC2893879. 20425186 PMC2893879

